# An update on articaine use in dentistry

**DOI:** 10.6026/973206300220102

**Published:** 2026-01-31

**Authors:** Genesis Hurtado, Navdeep Kaur, Visali Priyankitha Bomidi, Ashmita Kaur Kohli, Tayyaba Hamid, Fatima Zahid

**Affiliations:** 1 Department of Human Anatomy, School of Dentistry, Central University of Venezuela, Caracas, Venezuela; 2 Department of Dental Surgery, Sri Guru Ram Das Institute of Dental Sciences & Research, Amritsar, Punjab, India; 3 Department of Dental Surgery, Department of Oral and Maxillofacial Surgery, Sibar Institute of Dental Sciences, Guntur, Andhra Pradesh, India; 4 Department of Dental Surgery, I.T.S Dental College, Ghaziabad, Murad Nagar, Ghaziabad, Uttar Pradesh, India; 5 Department of Dental Surgery, Lahore Medical and Dental College, Lahore, Punjab, Pakistan; 6 Department of Dental Surgery, University of Lahore, Lahore, Punjab, Pakistan

**Keywords:** Articaine, dental anesthesia, infiltration, paresthesia, amide

## Abstract

Articaine hydrochloride is considered a highly effective and reliable local anesthetic in dentistry owing to its unique pharmacological characteristics. Its thiophene ring and ester group enhance lipid solubility, ensuring rapid tissue diffusion and efficient hydrolysis that limit systemic toxicity. The 4% formulation offers quicker onset, extended pulpal and soft tissue anesthesia and superior bone penetration compared with lidocaine, mepivacaine, and prilocaine. Recent advancements such as buffered formulations, antibacterial modifications and needle-free systems have further broadened its potential applications. This contributes to high procedural efficiency, improved patient comfort and prolonged anesthetic effects.

## Background:

Articaine is a short-acting, intermediate-potency amide local anaesthetic with a thiophene ring substitute for the benzene ring found in other amides, which increases its lipid-solubility and promotes rapid tissue diffusion [1]. It binds to the alpha subunit of the voltage-gated sodium channels, exerting a local anesthetic effect by preventing the inflow of sodium through the membrane and thereby inhibiting nerve conduction [2]. Pharmacokinetically, articaine exhibits high plasma protein binding (approximately 94%), which maintains stable plasma levels, prolongs its presence in the bloodstream, and limits the unbound fraction (6%) which is available for diffusion into tissues and eventual metabolism [3]. Unlike other amide anesthetics, the molecular structure of articaine contains an ester group, which is hydrolyzed in the blood to form an inactive metabolite, articainic acid, 75% of which is excreted unchanged, while the rest is metabolized in the kidneys to articainic glucuronide [3]. Due to its low systemic toxicity and higher efficacy, articaine can be used in higher concentrations than other amide local anesthetics [4]. Therefore, it is of interest to describe the role of articaine in dentistry.

## Comparative outcomes: Articaine versus other local anesthetics:

[1] Better diffusion and bone penetration: The thiophene ring enhances the diffusion ability of articaine across soft and hard tissues. This makes articaine better for infiltration anesthesia in the jaw, where other anesthetics often need nerve blocks [5, 6].

[2] Faster onset time: Articaine has a rapid onset of action compared to lidocaine and mepivacaine. This improves procedure efficiency and patient comfort [6, 7].

[3] Longer duration of action and lower need for supplemental injections: Articaine provides extended anesthesia compared with lidocaine, mepivacaine, and prilocaine, with 90 minutes of pulpal anesthesia and 230 minutes of soft tissue anesthesia for 4% articaine (with epinephrine) in an Inferior Alveolar Nerve Block (IANB) [8]. Due to its potency and diffusion, articaine often requires fewer supplemental injections than lidocaine [9].

[4] Better metabolism and clearance: Unlike other amide anesthetics, articaine is metabolized by both the liver and plasma esterases. This results in expedited systemic clearance and decreased toxicity risk, particularly in patients with hepatic impairment [10].

[5] Higher risk of paresthesia: Articaine is linked to a greater frequency of paresthesia and neurotoxicity compared to lidocaine or prilocaine [8, 11].

## Recent evidence on clinical efficacy in dental practice:

A 2021 meta-analysis of 22 trials evaluated the effectiveness of 4% articaine compared to 2% lidocaine for mandibular and maxillary block and infiltration anesthesia in patients with irreversible pulpitis. The study found that 4% articaine was 1.37 times more effective for mandibular pulpal anesthesia through infiltration and 1.06 times more effective for maxillary buccal infiltration [12]. A recent clinical trial evaluated 113 adults between 18 and 65 years old who had irreversible pulpitis in their molars. It assessed the effectiveness of 4% articaine and 2% lidocaine, and whether additional anesthesia was required when administered via Inferior Alveolar Nerve Block (IANB) and buccal infiltration. Pain levels were recorded at multiple time points using a Visual Analog Scale (VAS). Articaine demonstrated a higher success rate, especially with buccal infiltration (74% versus 57%, p = 0.03), as well as lower VAS for pain during access. In addition to this, fewer supplemental injections were needed with articaine [1]. In Pediatric dentistry, a randomized clinical trial published in 2023 compared 4% articaine with Inferior Alveolar Nerve Block (IANB), 4% via buccal infiltration (BI), and 2% lidocaine via Inferior Alveolar Nerve Block (IANB) in 27 children aged 8-12 years with molar incisor hypomineralization. The results showed that 4% articaine administered via Inferior Alveolar Nerve Block (IANB) yielded significantly lower pain scores during root canal access opening and instrumentation compared to 4% articaine (BI) or 2% lidocaine (IANB) [14]. A 2021 systematic review and meta-analysis published in the British Dental Journal examined the safety and effectiveness of 4% articaine with epinephrine compared to 2% lidocaine with epinephrine for routine dental procedures. It showed 2.17 times higher anesthetic success rate than lidocaine, and significantly, none of the studies reported anesthesia-related systemic side effects. Articaine was rated safe, effective, and efficient in patients of all ages in routine dental procedures [15].

## Safety and neurotoxicity considerations:

Although the safety and efficacy of articaine in dental procedures have been well established, paresthesia remains the most frequently reported complication, particularly after inferior alveolar nerve block (IANB) in non-surgical procedures [15]. While the absolute incidence of neurotoxicity is exceedingly low, the overrepresentation of paresthesia cases associated with 4% articaine is evident from retrospective clinical studies, compared to its market share [12]. Possible contributors to paresthesia may include the thiophene ring, higher concentration, and clinical factors like injection technique or nerve trauma. Initially, toxicity was attributed to the higher concentration of articaine, rather than intrinsic neurotoxic ability. Later experimental studies have shown that articaine is not intrinsically more cytotoxic, and in many cases, its tissue toxicity profile is equal to or lower than that of other amide anesthetics [17]. The dual metabolism of articaine results in a short half- life of approximately 20 minutes. Even though the vasodilatory effect of articaine could increase its systemic absorption, the rapid clearance prevents it from causing systemic toxicity [1]. All dental local anesthetics can exhibit neurotoxicity, depending on concentration and exposure time [18]. Clinical confounders, such as operator experience, mechanical trauma from the needle, and the increased vulnerability of the lingual nerve, also complicate the interpretation of reported cases. Most non-surgical paresthesia cases involve the lingual nerve, likely influenced by needle trauma, hematoma, fascicular patterns, or local tissue effects, rather than the anesthetic agent itself [19]. Randomized controlled trials and efficacy studies have found no significant increase in paresthesia with articaine compared to lidocaine. Articaine and lidocaine were found to produce similar hemodynamic responses during infiltration anesthesia [18]. A systematic review and meta-analysis reported no cases of permanent paresthesia. It concluded that articaine 4% is not neurotoxic and demonstrates better success than lidocaine in both infiltration and mandibular anesthesia [14]. Although articaine is a safe and effective local anesthetic, clinicians must remain aware of the extremely rare risk of long-term sensory disturbances. Further prospective studies using standardized reporting protocols are required to define these risks [15]. While some manufacturers suggest avoiding articaine in children under 4, studies have shown it is efficacious and safe when properly dosed (5 mg/kg). Articaine can be safely used in elderly patients with dose adjustments for underlying conditions. Although articaine is classified as FDA class C in pregnant women, it may be used during dental emergencies [20]. Recent reviews emphasize that articaine offers rapid onset, effective bone diffusion, and high anesthetic success rates. Although concerns have been raised regarding a potential association between 4% articaine and paresthesi, particularly following inferior alveolar nerve blocks, current evidence does not support a causal relationship. Overall, when administered with appropriate technique and dosing, articaine is regarded as a safe and effective local +anesthetic [21].

## Pediatric and special population applications:

## New insights:

## Pediatric populations:

Articaine has demonstrated effective pulpal and palatal anesthesia, including with the buccal infiltrations, in children aged 5-18 years, as reported in two studies in 2021 and 2025 [22, 23]. When compared with lignocaine for use in complex procedures like surgical endodontics, it was found to have better pain control, a lowered heart rate, and increased diastolic blood pressure, which led to a positive emotional response and pain relief in children [24]. A meta-analysis completed in 2023 on articaine usage in the pediatric population showed no probability of adverse reactions like postoperative pain, soft tissue injury, and edema [25]. Further, a randomized controlled trial in 2023 found a significant reduction in post-treatment pain and anxiety with articaine, used for intraoperative analgesia in children during dental rehabilitation under general anesthesia [26]. Although articaine is widely used in pediatric dentistry, it is recommended to exercise caution when using it in children under 4 years. Young children are more susceptible to toxic reactions because of their smaller body size and proportions [27]. The American Academy of Pediatric Dentistry (AAPD) guidelines recommend thorough documentation of type, dosage, and site of anesthesia, as well as monitoring for systemic effects [28].

## Pregnant and lactating women:

A literature review addressed dentists' reluctance to treat pregnant patients, emphasizing that delayed dental care will adversely affect both mother and child. It highlighted the safety of FDA category B and C local anesthetics, where routine drugs of category higher than C are commonly used in obstetrics, including during the first trimester. Additionally, this study indicates that articaine is a preferred anesthetic during pregnancy due to its established safety profile and widespread global usage [29]. Articaine may pass into the breast milk because of its high lipid solubility, causing a concern for breastfeeding babies [30]. A recent clinical lactation study reported that a 68 mg dose of 4% articaine via supraperiosteal infiltration reached its peak levels in breast milk at 0.25 hours and only 1.12 µg was secreted over 3 hours. This data is significantly below the maximum acceptable dose of 5mg/kg in children, suggesting that breastfeeding can be safely resumed 1 to 3 hours following articaine injection [31].

## Cardiovascular patients:

A study conducted before and after dental implant surgery demonstrated that the injection of 4% articaine with 1:100,000 epinephrine did not significantly affect blood pressure or heart rate [32]. A study conducted in 2024 showed that 4% articaine with adrenaline had no possible effect on the T wave in the Electrocardiogram (ECG) [33]. In addition, a double-blind trial demonstrated that the safe administration of articaine in conjunction with reduced epinephrine doses in hypertensive patients showed no significant cardiovascular differences compared with normotensive populations [34].

## Emerging research directions and future implications:

Local anesthetic solutions are typically made acidic to enhance solubility and stability. Formulations with vasoconstrictors contain sulfite preservatives, which lower the pH further. This increased acidity can delay the onset and cause more burning or stinging during injection. Studies have shown that buffering articaine improves patient comfort, accelerates onset, and enhances its effectiveness and duration, working well with both 1:100,000 and 1:200,000 epinephrine. While buffering local anesthetics is common in medicine, it is comparatively difficult in dentistry due to the cartridge design. A technology focused on the syringe design featuring separate chambers for the buffering agent and local anesthetic solution has the potential to further simplify chairside buffering, thereby improving workflow [35]. Local anesthesia (LA) carries a risk of post-injection infections. Articaine and lidocaine have been found to inhibit some bacterial growth, but researchers are focusing on developing articaine derivatives that have more potent antibacterial activity at the Minimum Inhibitory Concentration (MIC). Articaine-15 (AT-15) has been found to significantly inhibit the colony growth of P. gingivalis and E. coli at a concentration of 10 - 20 mg/ml [36, 37]. Prospective directions in articaine research might also include customizing the formulations of articaine for specific procedures. For instance, employing needle-free drug delivery systems may improve pain management and decrease the risk of intraoperative complications, making them the drugs of choice in patients facing dental anxiety or systemic health challenges [38]. While lignocaine is widely regarded as the gold standard local anaesthetic, evidence indicates that 4% articaine provides a more rapid onset and a longer duration of action, supporting its consideration as an effective alternative [39]. Furthermore, the use of articaine as an anesthetic nanomedicine is being explored. A multiscale modelling study published in 2023 by Mirjanic *et al.* analyzed the new drug delivery system, such as integrated graphene- based systems, may enable a sustained drug release and increase the duration of pain relief in dental procedures [40].

## Conclusion:

Recent studies support the clinical benefits of using 4% articaine over other anesthetics during various dental procedures for more effective and less painful anesthesia in both adult and pediatric patients. It also has a lower need for supplemental injections and a safe anesthetic profile. Articaine can be a preferred anesthetic for modern dental practice, provided clinicians adhere to aseptic working techniques and remain aware of dosage-related complications.

## References

[1] Oertel R (1997). Clin Pharmacokinet..

[2] Wang GK (2009). J Membr Biol..

[3] Vree TB, Gielen MJM (2005). Best Pract Res Clin Anaesthesiol..

[4] Huang N-C (2025). J Dent Sci..

[5] Kanaa MD (2006). J Endod..

[6] The Luo W (2022). Medicine (Baltimore)..

[7] Costa CG (2005). Quintessence Int..

[8] Haas DA (2002). J Can Dent Assoc..

[9] Brandt RG (2011). J Am Dent Assoc..

[10] Malamed SF (2001). J Am Dent Assoc..

[11] Gaffen AS, Haas DA (2009). J Can Dent Assoc..

[12] Miglani S (2021). PeerJ..

[13] Sattar R (2025). J Health Wellness Clin Res..

[14] Thomas AM (2023). J Indian Soc Pedod Prev Dent..

[15] Martin E (2021). BDJ Open..

[16] Agwani K (2022). Int J Early Child Spec Educ..

[17] Hopman AJG (2017). Br Dent J..

[18] Baroni DB (2013). Acta Odontol Scand..

[19] Pogrel MA (2003). J Am Dent Assoc..

[20] Gulenko OV, Vasil'ev YL (2021). Stomatologiia (Mosk)..

[21] Saraghi M, Hersh EV (2022). Gen Dent..

[22] Bahrololoomi Z, Rezaei M (2021). J Dent Anesth Pain Med..

[23] Jain K (2021). J Indian Soc Pedod Prev Dent..

[24] Che Y (2024). Perioper Med (Lond)..

[25] Li L, Sun DL (2023). J Clin Pediatr Dent..

[26] Janiani P (2023). J Popul Ther Clin Pharmacol.

[27] Saket A (2024). J Popl Ther Clin Pharmacol..

[28] https://www.aapd.org/globalassets/media/policies_guidelines/bp_pain.pdf.

[29] Mitchell J (2020). Anaesthesia..

[30] Liu SP (2025). J Am Dent Assoc..

[31] Manautou MA, Mayberry ME (2023). J Evid Based Dent Pract..

[32] https://www.researchgate.net/publication/266802992.

[33] Pokel C (2024). Int J Dent..

[34] https://www.ajol.info/index.php/njmde/article/view/290801.

[35] https://decisionsindentistry.com/article/perspectives-buffered-articaine-dentistry.

[36] Kaewjiaranai T (2018). J Dent Anesth Pain Med..

[37] Tan Y (2025). J Oral Microbiol..

[38] Qian S (2025). J Pain Res..

[39] Manas A (2025). Bioinformation..

[40] Mirjanic V (2024). J Mol Liq..

